# Recovery of Aconitic Acid from Sweet Sorghum Plant Extract Using a Solvent Mixture, and Its Potential Use as a Nematicide

**DOI:** 10.3390/life13030724

**Published:** 2023-03-08

**Authors:** K. Thomas Klasson, Yunci Qi, Gillian O. Bruni, Tristan T. Watson, Bretlyn T. Pancio, Evan Terrell

**Affiliations:** 1 Southern Regional Research Center, Agricultural Research Service, United States Department of Agriculture, New Orleans, LA 70124, USA; 2 Department of Plant Pathology and Crop Physiology, Louisiana State University, Baton Rouge, LA 70803, USA; 3 Oak Ridge Institute for Science and Education Research Program at USDA, Oak Ridge, TN 37831, USA

**Keywords:** acetone:butanol:ethanol, solvent extraction, process design and economics, *Caenorhabditis elegans*, *Meloidogyne incognita*

## Abstract

*Trans*-aconitic acid (TAA) is naturally present in sweet sorghum juice and syrup, and it has been promoted as a potential biocontrol agent for nematodes. Therefore, we developed a process for the extraction of aconitic acid from sweet sorghum syrup. The process economics were evaluated, and the extract was tested for its capability to suppress the motility of the nematodes *Caenorhabditis elegans* and *Meloidogyne incognita*. Aconitic acid could be efficiently extracted from sweet sorghum syrup using acetone:butanol:ethanol mixtures, and it could be recovered from this solvent with a sodium carbonate solution, with an overall extraction and recovery efficiency of 86%. The estimated production cost was USD 16.64/kg of extract and this was highly dependent on the solvent cost, as the solvent was not recycled but was resold for recovery at a fraction of the cost. The extract was effective in reducing the motility of the parasitic *M. incognita* and causing over 78% mortality of the nematode when 2 mg/mL of TAA extract was added. However, this positive result could not conclusively be linked solely to TAA. An unidentified component (or components) in the acetone:butanol:ethanol sweet sorghum extract appears to be an effective nematode inhibitor, and it may merit further investigation. The impact of aconitic acid on *C. elegans* appeared to be entirely controlled by pH.

## 1. Introduction

Aconitic acid (prop-1-ene-1,2,3-tricarboxylic acid) is the most abundant C6 organic acid that accumulates in sugar crops such as sugarcane and sweet sorghum. Aconitic acid functions as a chemical precursor and is intermediate to several value-added chemicals and products; it was listed as a top 30 value-added chemical by the U.S. Department of Energy [1]. There is considerable interest in using aconitic acid as a chemical precursor or reactant with its three reactive carboxylic acid groups. It is especially attractive, since it can be inexpensively sourced from renewable agricultural byproducts such as sugarcane molasses and sweet sorghum syrup to produce bio-based products and chemicals with various properties that we recently reviewed [2]. For instance, aconitic acid has been used in the production of polyesters, hyper-branched polyesters, polymers, and as a chemical crosslinker, plasticizer, and grafting agent [3,4,5,6,7].

In addition to its numerous chemical applications, aconitic acid has several biological roles and functions that were recently summarized by Bruni and Klasson [2], and it has many bioactive functions in specific plants and microorganisms, including its impact on nematodes [8,9,10]. Clarke and Shepherd [8] reported that in a study of 444 inorganic and organic compounds, *trans*-aconitic acid was one of 45 compounds that stimulated the eggs of the potato cyst nematode *Heterodera rostochiensis* (now named *Globodera rostochiensis*) to hatch at 3 mM (0.500 mg/mL) but it was not among the strongest stimulants. It was even less effective in stimulating *H. schachtii* to eggs to hatch [8]. Contrary to these findings of being a stimulant, Du et al. [9] found that aconitic acid at 0.5 mg/mL killed approximately 83% of root knot nematode *Meloidogyne incognita*, and it was 92% effective against this nematode at 1.0 mg/mL. In separate research, when studying the impact of *Canavalia ensiformis* (jack bean) seed extract on *M. incognita*, Rocha et al. [10] concluded that *trans*-aconitic acid was one of the organic acid constituents in the seed extract that acted as a nematicide and that paralyzed 98% of *M. incognita* at 0.5 mg/mL. *Cis*-aconitic acid was less effective (65%), but it still showed bioactivity.

One potentially significant natural source of aconitic acid is from sugar crops, such as sugarcane and sweet sorghum. Aconitic acid accumulates in these plants through the citrate branch of the tricarboxylic acid (TCA) cycle. In sugarcane, aconitic acid can account for roughly 0.1 to 0.5% of stalk juice [11], while in cane molasses, its concentration can be as high as 1 to 5% of dissolved solids [12]. Sweet sorghum syrup has been reported to similarly contain about 1% aconitic acid [13], with juice concentrations varying between roughly 0.2 and 0.6% for sweet sorghum, depending on the cultivar [14]. In one study on the recovery of polymerization grade aconitic acid from cane molasses, Kanitkar et al. [12] reported yields of 34 to 69% when using ethyl acetate as an extractive solvent, with aconitic acid purities of up to 99.9%. These authors also reported yields and purities of 62% and 99.9%, respectively, for the recovery of aconitic acid from fermented molasses with ethanol as a co-product. Other solvents reported in the literature for aconitic acid extraction from molasses include methyl-isobutyl ketone, methyl ethyl ketone, tributyl phosphate, amines, xylene, hexane, chloroform, and alcohols [11,12,15].

When comparing three processes for aconitic acid recovery from molasses (methanol precipitation; solvent extraction; ion exchange), Regna and Bruins [16] reported that solvent extraction appears to be the most economical, with a (producer price index adjusted, [17]) cost per kg of aconitic acid in the range of USD 6.09–USD 8.61, compared to USD 7.20–USD 9.63 for methanol precipitation and USD 7.35–USD 9.94 for ion exchange. While methyl-ethyl-ketone was the primary organic solvent used in their work, the authors reported that other organic solvents (specifically butanol) could be easily substituted, with alcohol solvents having the advantage of being derivable as a product from molasses fermentation. Aconitic acid precipitate recovery from sweet sorghum juice has been reported [18,19] during clarification in juice processing, with the addition of lime and calcium chloride, where the primary goal is the improvement of sucrose crystallization. However, because of general similarities between cane molasses and sorghum syrup, the recovery of aconitic acid (where present) from these low-cost feedstocks using organic solvents should be comparably feasible.

As butanol is one of the solvents that resulted in significant aconitic extraction [11], this research focused on butanol mixtures. Butanol can be produced commercially via fermentation, but it is co-produced with acetone, ethanol, and minor quantities of organic acids [20,21,22]. The separate recoveries of acetone, butanol, and ethanol from the fermentation broth have been studied extensively [23,24,25,26,27]. In a typical acetone:butanol:ethanol (ABE) fermentation [28], the molar ratio of the products is 0.39:1:0.12 (A:B:E), but it can have other compositions as well, depending on many factors (e.g., head-space H_2_ and CO, electron sinks, etc.) [29,30,31,32]. The purpose of this study was to investigate the applicability of using mixtures of acetone:butanol:ethanol for the extraction of aconitic acid from sweet sorghum syrup. This could be seen as an intermediate step to using the sweet sorghum sugars for other applications; i.e., first separating aconitic acid and then using the sugars. Additionally, it can be seen as an intermediate step for recovering and purifying acetone, butanol, and ethanol from a fermentation; i.e., the complete separation of acetone, butanol, and ethanol may not be needed for aconitic acid extraction, and it could be delayed after it has been used for aconitic acid extraction. Another goal of the study was to evaluate the capacity of the aconitic acid to inhibit the motility of free-living nematodes and parasitic nematodes. *Caenorhabditis elegans* was chosen as it does not require a permit, and because the organism is easy to store, maintain, and culture [33]; and *Meloidogyne incognita* was chosen as others reported its susceptivity to aconitic acid [9,10].

## 2. Materials and Methods

Commercially available sweet sorghum syrup (Village Valley Sweet Sorghum Syrup, Delta BioRenewables LLC (Memphis, TN, USA) and a non-commercial batch of sweet sorghum syrup from Delta BioRenewables [13] were used to determine the optimal aconitic acid extraction conditions, and to create a crude extract for nematode testing. The non-commercial batch was chosen for the final crude extract preparation of aconitic acid, as it contained higher levels of initial aconitic acid than the commercial product.

### 2.1. Aconitic Acid Extraction

Solvent ratios of acetone:butanol:ethanol used during the extraction optimization are shown in Table 1. They were chosen to represent a range of acetone:butanol ratios, representative of the products from an ABE fermentation, which can be produced at different ratios [28,29,30,31,32].

The ratios of organic solvent (ABE mixtures) to diluted sweet sorghum syrup in the extractions were 1, 2, 3, 3.5, and 4 (g organics/g syrup). At least four experiments were performed for each extraction condition. If the standard errors of the extraction efficiency were deemed as large, additional extraction experiments were performed. Statistical differences between the results were determined using Tukey’s Honest Significant Difference (HSD) procedure [34].

Before extraction, sweet sorghum syrup was diluted to 50 °Brix (~50% dissolved solids), had its pH adjusted to pH 2.0 using 0.2–4 M H_2_SO_4_ (~0.03–0.04 mL/g syrup), and was analyzed for sucrose, glucose, fructose, and aconitic acid. The diluted syrup and organics mixture were combined in 15 mL or 50 mL centrifuge tubes (~2/3 full) and incubated for 30 min at 50 °C, after which time the mixture was briefly removed from the incubator and shaken vigorously every 10 min for 60 min. After extraction, the tubes were centrifuged at 3000 rpm (2100× *g*) for 5 min. The organic layer was removed, and the aqueous phase was analyzed for sugars and aconitic acid after a 10-fold dilution. The weights of each phase before and after extraction were recorded and used in the calculations.

For the final extractions to generate a crude extract of aconitic acid, all of the same procedures were followed as previously described, using an acetone:butanol:ethanol (ABE) mixture of 0.19:0.74:0.07 (wt:wt:wt), and a ratio of 2.5 (g organics/g diluted syrup). The back-extraction was performed using 0.4 M Na_2_CO_3_ and a ratio of 6 (g organics/g salt solution). To remove some of the solvents contained in the extract, the extract was placed under a vacuum on a rotary evaporator (Model R-200, Buchi Corp., New Castle, DE, USA) at 60 °C for 30 min, which resulted in an 11% mass loss.

Sucrose, glucose, fructose, acetone, butanol, ethanol, and aconitic acid were analyzed using high performance liquid chromatography using the methods and equipment previously described [13]. Typical chromatograms from sample analysis have been provided in the Supplementary Materials.

### 2.2. Nematicidal Assay for Caenorhabditis Elegans

Initial nematocidal assay experiments were carried out with *C. elegans*, a free-living model nematode [35,36] as it does not require a permit and the organism is easy to store, maintain, and culture [33]. *Escherichia coli* strain OP50-1, obtained from the Caenorhabditis Genetics Center (CGC, University of Minnesota, Minneapolis, MN), was cultured in Luria-Bertani (LB) medium (Becton Dickinson, Sparks, MD, USA). *C. elegans* strain N2, obtained from CGC, was routinely propagated on Nematode Growth Medium (NGM) agar [37] seeded with *E. coli*. Age-synchronized worms were obtained as previously described [37]. Briefly, gravid *C. elegans* were collected from NGM agar plates with water and treated with sodium hypochlorite to obtain the eggs, which were resuspended in S buffer [38] and allowed to hatch into L1-stage larvae by incubating overnight at 20 °C. The L1-stage larvae were collected in a pellet by centrifuging at 1200× *g* for 3 min and re-suspending the pellet in fresh S buffer with *E. coli* for 24 h at 20 °C to obtain approximately early L3-stage worms. The number of worms per mL of S buffer was estimated by spotting 10 droplets of 10 µL on a plate and averaging the number of worms per drop.

The nematocidal assay protocol was adapted from those described by Du et al. [9] and Watson [39]. After counting the number of worms per mL, the appropriate volume of worm suspension was centrifuged and resuspended with fresh S buffer to obtain approximately 1000 worms per mL. For experiments with aconitic acid solution in water, S buffer was used, while the remaining experiments used 0.1 M Na_2_CO_3_ (pH adjusted to the same as TAA solution or extract). In a sterile 96-well plate, 50 µL volumes of worm suspension were aliquoted (approximately 50 worms per well), followed by the addition of 50 µL of aconitic acid solution. *Trans*-aconitic acid (>98% purity) was obtained from Sigma-Aldrich (St. Louis, MO, USA). After the plates were incubated at 20 °C for 48 and 72 h, the number of motile and immotile worms were counted under a microscope. As controls, worms were treated with 4 mg/mL fluopyram (Velum Prime, Bayer Crop Science, Whippany, NJ, USA). Assays were performed in triplicate, and the statistical differences between the results were determined using Tukey’s HSD procedure.

### 2.3. Nematode Assay for Meloidogyne Incognita

Motility assays for *M. incognita* (originally isolated from sweet potato) were performed as previously described [39]. Briefly, plate wells (a 6-well microplate) were filled with 4 mL of aqueous solutions of each treatment solution. Approximately 150 J2-stage nematodes were suspended in 10 µL of water and introduced into each well. Nematode motility and mortality were recorded at 48 and 72 h post-inoculation under a stereomicroscope. The entire experiment was performed twice, with each treatment performed in triplicate (i.e., n = 6). Fluopyram (4 mg/mL) was included as a positive control for the inhibition of motility. Significant differences were evaluated using Turkey’s HSD procedure.

### 2.4. Process Design

The process design, sizing of equipment, and cost estimation were performed using SuperPro Designer, Version 11.2 (Intelligen, Scotch Plains, NJ, USA). The annual production of the sweet sorghum syrup, containing 65% (wt/wt) total dissolved solids (mostly sugars) and 1.2% aconitic acid was assumed to be 8,000,000 kg. This is representative of a small-sized but commercial production of syrup [40]. While this amount is generated during a short harvesting season (approximately 4 months), it was considered to be stored and available at a flow rate of 1010 kg/h for 7920 h. The syrup was heated, diluted to 50 °Brix (~50% dissolved solids), and pH adjusted to pH 2.5 before extraction with an acetone:butanol:ethanol (ABE) mixture (19%:74%:7%); a mixture found to be optimal in the preliminary studies. The organic and aqueous phases were separated, after which the aconitic acid in the organic phase was cooled and back-extracted with sodium carbonate (0.4 M). This was followed by flash evaporation to partially remove additional solvents for ABE recovery. The spent organic phase (containing most of the ABE) was processed for the recovery of ABE for extraction reuse (not modeled here). The ABE recovery was recently reported elsewhere [23,24,25,26,27]. The aqueous phase, containing approximately 50% sugars, can be used for an ABE fermentation (not modeled here). Any remaining unused solvent streams are passed on to solvent separation and recovery. The value of the sugar and ABE stream was valued at USD 0.46/kg of sugar [41] and 10% of the price of ABE. The prices of acetone, butanol, acetone, sodium carbonate, and sulfuric acid were obtained from Intratec [42]. All prices were adjusted to 2022 prices using the producer price index [17]. Other costs such as materials, labor, facilities, etc., were obtained directly from the SuperPro Designer software.

## 3. Results and Discussion

### 3.1. Aconitic Acid Extraction

The commercial syrup contained 5.1 g/kg, and the non-commercial syrup contained 12.0 g/kg of aconitic acid. After dilution to 50 °Brix and pH adjustment, the commercial and non-commercial syrups contained 3.3 and 9.3 g/kg of aconitic acid, respectively. The lower level of aconitic acid in the commercial syrup was likely due to additional filtration used with commercial syrup production. It has been reported that sweet sorghum syrup contains both soluble and insoluble aconitic acid [18]. These concentrations are typical, as others have reported values of 0.26–4.8 g/L in the sweet sorghum juice [14,43,44], and 10–11 g/kg of aconitic acid in the sweet sorghum syrup [13].

**Figure 1:**
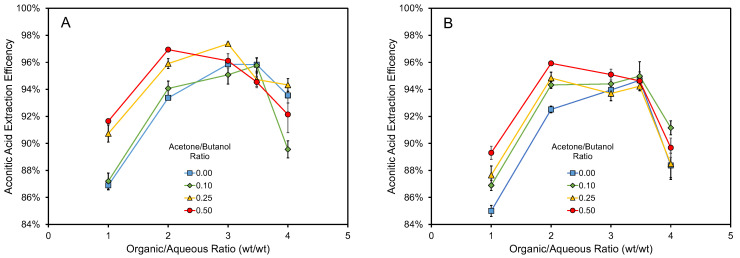
Aconitic acid extraction efficiency using different ABE mixtures and different solvent:syrup ratios for commercial syrup (**A**) and non-commercial syrup (**B**). The efficiency was calculated by the difference in the amount of aconitic acid present in the syrup before and after solvent extraction, accounting for weight changes to the phases after extraction. Error bars represent standard errors.

The extraction efficiency of the solvents to extract aconitic acid from the syrups is shown in Figure 1. As noted, the maximum extraction efficiency was obtained using an approximately 2–3.5 organics:syrup phase weight ratio. A slightly higher extraction efficiency was obtained when extracting aconitic acid from the commercial syrup than when extracting aconitic acid from the non-commercial syrup. Overall, 92–96% aconitic acid was extracted with the 2–3.5 organics:syrup ratio; and generally, the extraction efficiency increased as the acetone:butanol ratio in the solvent phase increased (Figure 1). The results compared well with Gil Zapata [11], who tested solvents for the extraction of aconitic acid from fermented and distilled sugarcane juice and molasses. He reported an extraction efficiency of 90% when pure butanol was used at pH 2.0 with an organic:aqueous ratio of 3.5. Others [15] have obtained a high extraction efficiency using amine- or phosphorous-based solvent systems, in combination with chloroform, xylene, hexane, etc. The best solvent systems extracted over 98% of the aconitic acid from sugarcane molasses at pH 1.5–1.6 [15].

**Figure 2:**
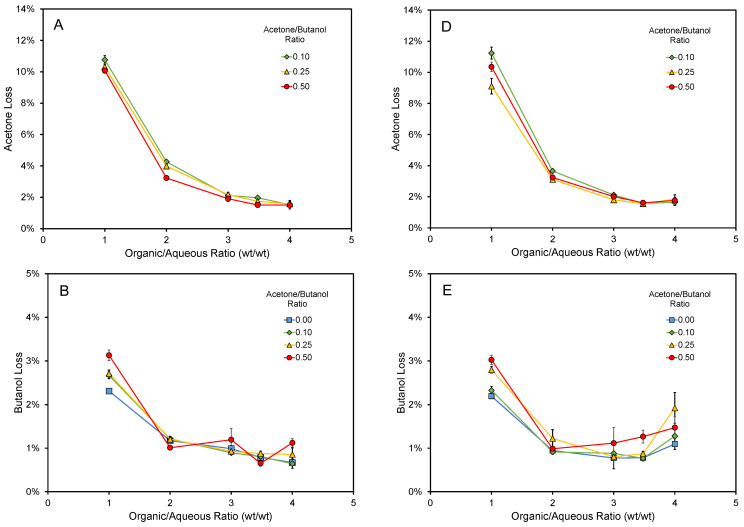

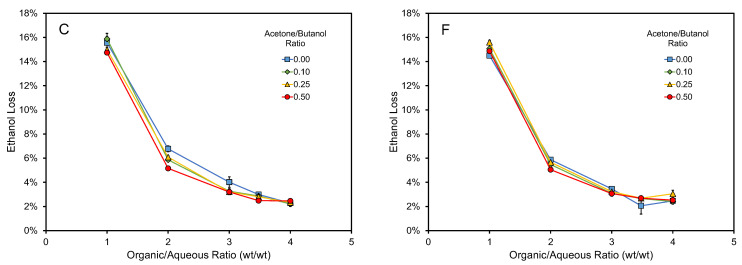
Solvent loss to the syrup phase during extraction with different ABE mixtures and different solvent:syrup ratios. Acetone, butanol, and ethanol losses for commercial syrup are shown in (**A**), (**B**), and (**C**), respectively. Acetone, butanol, and ethanol losses for non-commercial syrup are shown in (**D**), (**E**), and (**F**), respectively. The loss was calculated using the difference of the solvent species present in the syrup before and after solvent extraction, accounting for weight changes to the phases after extraction. Error bars represent standard errors.

The amount of solvent lost to the spent syrup was also determined (Figure 2). Lower organics:syrup ratios led to a greater loss of solvent. At the optimal organics:syrup ratio for aconitic acid extraction (2–3.5), approximately 2–4%, 1%, and 2–6% of acetone, butanol, and ethanol were lost in the spent syrup, respectively (Figure 2). The acetone:butanol ratio did not impact on the solvent loss at the ranges studied.

To produce the extract used for nematode testing, larger quantities of the non-commercial sweet sorghum syrup, an acetone:butanol:ethanol mixture of 0.19:0.74:0.07 (wt:wt:wt), and an organic:syrup ratio of 2.5 (wt:wt) were used. This resulted in an extraction of 96% of the aconitic acid from the syrup. The back-extraction of the aconitic acid from the solvent phase was performed using 0.4 M Na_2_CO_3_ and a ratio of 6 (g organics/g salt solution). After solvent evaporation from the receiving salt solution and adjusting the pH to 6.5 with diluted sulfuric acid, the extract contained 45 g/L of aconitic acid. Overall, this represents 84% recovery of aconitic acid from the diluted syrup. The efficiency of Na_2_CO_3_ for the back-extraction of aconitic acid was also noted by Blinco et al. [15], who used 0.1–0.2 M Na_2_CO_3_ to back-extract 91–95% of aconitic acid from a solvent phase containing tributyl phosphate and the industrial solvent Shellsol that had previously been used to extract the aconitic acid from molasses.

### 3.2. Nematicidal Assays with C. elegans

**Figure 3:**
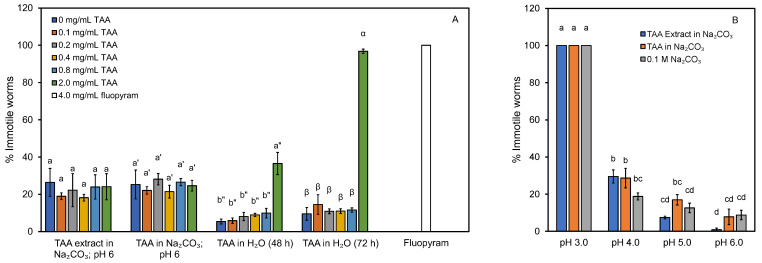
Nematicidal assays with TAA, TAA extract, and buffer against *C. elegans*. (**A**) The crude TAA extract, TAA in 0.1 M Na_2_CO_3_, and TAA solutions in water without pH adjustment. Fluopyram was used as control treatment. (**B**) The crude TAA extract, TAA in 0.1 M Na_2_CO_3_, and Na_2_CO_3_ control, adjusted to pH 3 to pH 6. Unless specified, the assays were carried out for 48 h. Percentage immotile includes dead worms. Significant differences between different treatments at the same condition in 3A and between any condition in 3B are represented by different letters above standard error bars.

The crude TAA extract was tested as a potential nematicide based on results previously reported by Du et al. [9]. The same results were obtained with the TAA extract and the TAA standard in Na_2_CO_3_ buffer at all TAA concentrations studied, resulting in approximately 22% immotile worms (Figure 3A, bar grouping 1 and 2). Less nematocidal activity was observed in assays with solutions of TAA in water, except in the case of 2 mg/mL TAA, where approximately 36% immotile worms were observed at 48 h (Figure 3A, bar grouping 3), which increased to 97% at 72 h (Figure 3A, bar grouping 4). Upon further investigations, it was determined that the extract preparation included neutralization with Na_2_CO_3_ and had a significant buffering capacity. While the buffering capacity was also present in the nematode growth medium, the pH was low (e.g., pH 2.7 with 2 mg/mL of TAA) in the final assay conditions when TAA in water was tested. The pH of the final assay solution was approximately pH 6 when the TAA extract was tested. Thus, it was speculated that pH may have a strong impact on the effectiveness of TAA against the nematodes.

The inhibitory activity of TAA against other organisms, e.g., the yeast *Saccharomyces cerevisiae*, has been reported to be pH-dependent [45]. This inhibition by undissociated organic acids is caused by an increase in transport into the cell, and a decreased internal cell pH when the organic acid dissociates and releases its protons [46]. Thus, the pH 2.7 of a 2 mg/mL solution of TAA in the assay could negatively impact on nematode viability/motility, causing the 36% and 97% immotile worms at 48 and 72 h, respectively (Figure 3A, bar groupings 3 and 4). Assays with TAA extract and TAA standard in 0.1 M Na_2_CO_3_ (0.1–2 mg/mL of TAA) adjusted to pH 6 showed the same nematocidal activities (Figure 3A, bar groupings 1 and 2) as in the buffer control (0 mg/mL TAA). Subsequent tests with buffered solutions of 2 mg/mL TAA (or buffer only) adjusted to pH 3.0 to pH 6.0 suggest that nematocidal activity of TAA may be entirely attributed to its acidity during assays (Figure 3B). This is supported by Khanna et al. [47], who reported an increased mortality of *C. elegans* of below pH 3.1–3.4, depending on the environment (salinity). It is important to note that a previous report did not study or list the pH in their nematode studies with TAA [9]. Note that the majority of aconitate is fully protonated below pH 2.71 (=pK_a1_) [45].

### 3.3. Nematicidal Assays with M. incognita

**Figure 4:**
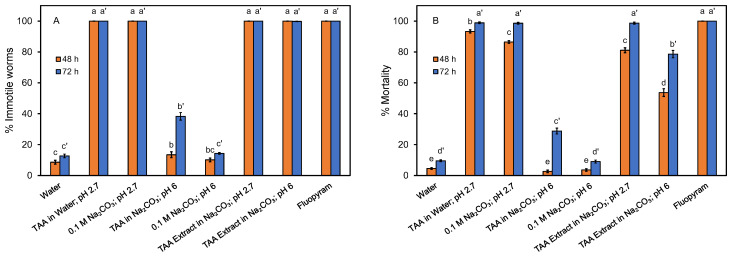
Nematicidal assays, mobility (**A**), and mortality (**B**), with crude TAA extract and standards versus *M. incognita*. All TAA solutions contained 2 mg/mL. Fluopyram (4 mg/mL) was used as a control. Percent immotile includes dead worms. Significant differences between treatment conditions (within each graph) are represented by different letters above standard error bars.

While the crude TAA extract did not appear to be an effective nematicide against *C. elegans*, a recent study suggested that TAA may affect free-living nematodes differently than parasitic nematodes such as *Meloidogyne incognita* [10]. As such, assays were performed to test the inhibitory activity of the crude TAA extract and a TAA standard, both diluted to 2 mg/mL and adjusted to pH 2.7 and pH 6, against the root knot nematode *M. incognita* (Figure 4). Nematode motility was inhibited, and mortality was high with TAA in water at pH 2.7. However, we found similar results with a Na_2_CO_3_ solution at pH 2.7 (without TAA). We found that the nematode was unaffected at 48 h and was modestly affected at 72 h by TAA in Na_2_CO_3_ at pH 6.0, but was unaffected by Na_2_CO_3_ at pH 6. This suggests that the impact on *M incognita* by TAA is pH-dependent. This finding contradicts a previous report that found 92.1% mortality of *M. incognita* with TAA (1 mg/mL, pH unknown) [9]. Interestingly, our study showed a significant inhibition from the TAA extract at both pH 2.7 and pH 6.0, which suggests that there are unidentified compounds in the extract that inhibit *M. incognita*. Further studies may explore this, and it is important to note that residual acetone, butanol, and ethanol (0.0, 0.5, and 0.4 mg/mL at 2 mg/mL TAA) in the crude extract may also affect nematodes. The results obtained at pH 2.7 may be less relevant, as this soil organism may not experience pH levels below pH 4 [48]. In conclusion, *M. incognita* motility and mortality were affected by the TAA extract, while *C. elegans* was not.

### 3.4. Process Economics of Aconitic Acid Extraction from Sweet Sorghum Syrup

**Figure 5:**
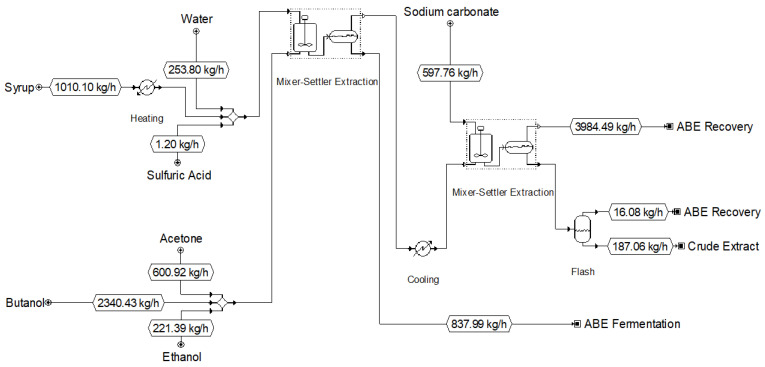
Process flow diagram of aconitic acid extraction and recovery from sweet sorghum syrup. The SuperPro Designer file is provided in the Supplementary Materials.

SuperPro Designer was used for design and process economics. The flow sheet of the process is shown in Figure 5. Various variations to the basic flow sheet were explored, primarily for heat recovery and heat management, but very little impact was noted on the final economic analysis.

The estimated equipment cost to produce 1,481,483 kg extract per year was USD 93,000, and the total capital investment was USD 3,211,000. The significant difference between these values is due to the initial purchase of raw materials (mainly ABE). A breakdown of the capital and operating cost is shown in Table 2. The estimated net cost of production would be USD 16.64/kg of extract. It is difficult to compare this cost compared the estimated production cost developed by Regna and Bruins [16] over 50 years ago. The estimated production cost is derived mainly from solvent (ABE) use, which was not recycled in the process and was only valued at 10% of its purchase cost. It is envisioned that the extract production would be part of an integrated biorefinery producing ABE from sweet sorghum syrup. In addition, the annual production volume is low for this highly specialized product, which does not have the economic benefit of a larger production scale.

## 4. Conclusions

Aconitic acid can be efficiently extracted from sweet sorghum syrup using acetone:butanol:ethanol mixtures. An aconitic acid extract can then be recovered from this solvent with a Na_2_CO_3_ solution, with an overall extraction and recovery efficiency of 86%. The estimated production cost was USD 16.64/kg of extract, and this was highly dependent on the solvent cost, as the solvent was not recycled but was resold for recovery at a fraction of the cost.

The extract, containing *trans*-aconitic acid (TAA), had little impact on the motility of the model nematode *C. elegans* when compared to chemically pure TAA or the pH buffered control. In conclusion, it was determined that the low pH effect of unbuffered TAA in the *C. elegans* nematocidal assay was responsible for increased motility reduction. The extract was effective in reducing the motility of the parasitic *M. incognita* and causing over 78% mortality of the nematode. However, this positive result could not be conclusively linked to TAA. Thus, in contrast to prior reports, we found that aconitic acid was not an effective inhibitor of the nematodes *C. elegans* and *M. incognita*. Finally, an unidentified component (or components) present in the acetone:butanol:ethanol sweet sorghum extract appears to be an effective inhibitor of *M. incognita*, and may merit further investigation.

## Figures and Tables

**Table 1:** Composition of acetone:butanol:ethanol solutions used in the extraction of aconitic acid from diluted sweet sorghum syrup.

Weight Fraction			Molar Fraction		
Acetone	Butanol	Ethanol	Acetone	Butanol	Ethanol
0.00	0.93	0.07	0.00	0.89	0.11
0.08	0.84	0.07	0.10	0.79	0.11
0.19	0.74	0.07	0.22	0.68	0.11
0.31	0.62	0.07	0.35	0.55	0.11

**Table 2:** Capital and operating cost for an extraction process to produce an aconitic acid extract.

Cost Item	Cost
Total Capital Investment	USD 3,211,000
Annual Operating Cost (AOC)	USD 29,059,000
Raw Materials	USD 26,475,000 (91% of AOC)
Labor	USD 2,108,000 (7% of AOC)
Facilities/Laboratory	USD 425,000 (1% of AOC)
Utilities	USD 51,200 (0.2% of AOC)
Credits (sugars and ABE)	USD 4,406,000 (15% of AOC)
Net Production Cost	USD 16.64/kg of extract

## Data Availability

The data presented in the figures are available from the corresponding author upon reasonable request.

## Supplementary Materials

The following supporting information can be downloaded at https://www.mdpi.com/article/10.3390/life13030724/s1, SuperPro Designer Process File: MDPI-Life-Klasson-2023.spf; Figure S1: Example of HPLC chromatogram with labeled peaks.
