# Supplementary material for: Recovery of Aconitic Acid from Sweet Sorghum Plant Extract Using a Solvent Mixture, and Its Potential Use as a Nematicide
Source: Life (Basel). 2023 Mar 8;13(3):724. doi: 10.3390/life13030724 (PMC10054008; doi:10.3390/life13030724)
Supplement: Supplementary file 1 [file life-13-00724-s001.zip › MDPI-Life-Klasson-2023.pdf]

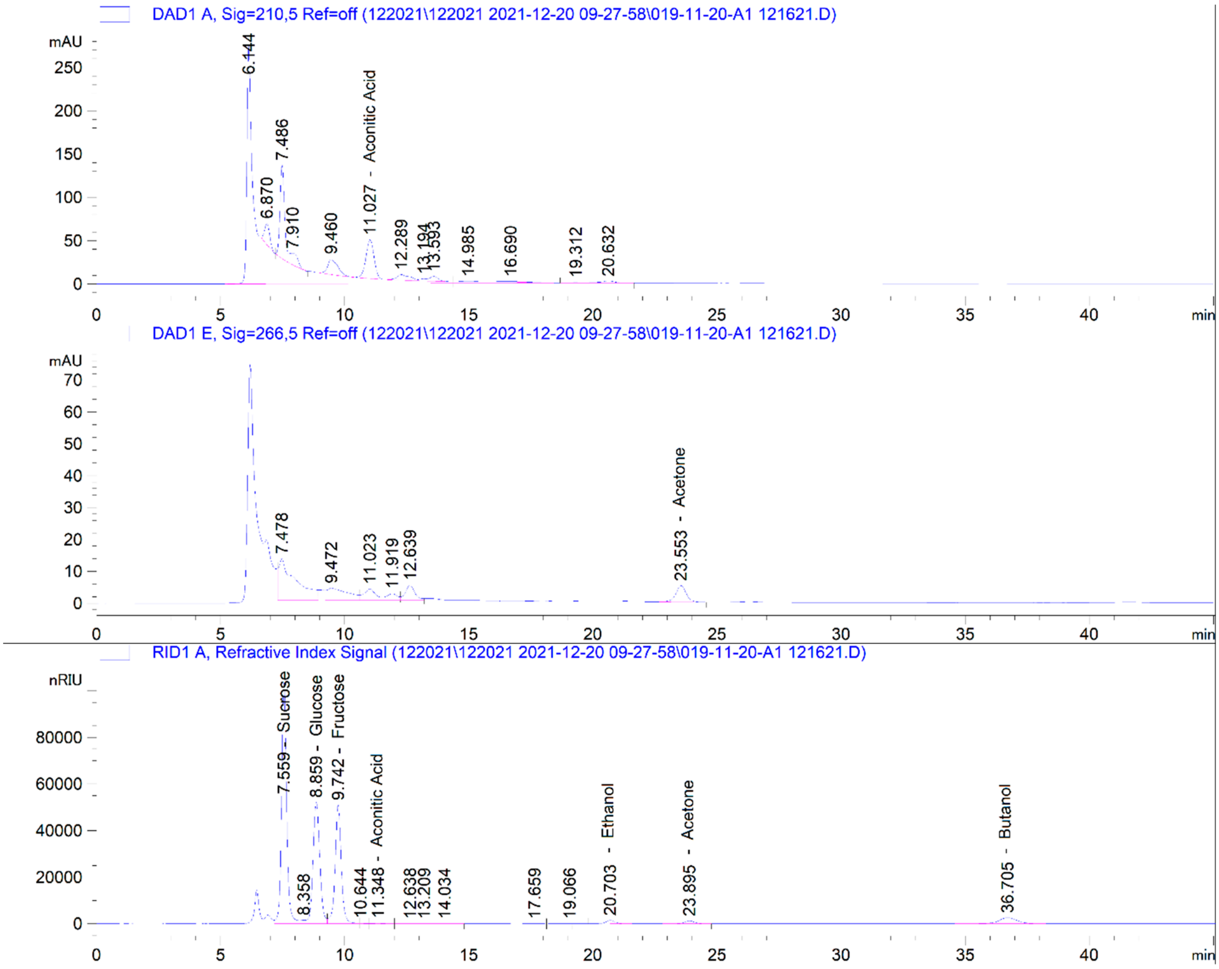

Figure S1. Example HPLC chromatogram with labeled peaks. Experimental Conditions: ABE extraction of commercial sweet sorghum syrup containing TAA. Organics/Syrup Ratio = 1.0 wt/wt. A/B/E Ratio = 0.08/0.84/0.07 (wt/wt/wt). HPLC conditions were described in reference 13, using RID and DAD (210 and 266 nm) sequential detectors.
